# A Review of Current Perspectives on Motoric Insufficiency Rehabilitation following Pediatric Stroke

**DOI:** 10.3390/healthcare12020149

**Published:** 2024-01-09

**Authors:** Hristina Colovic, Dragan Zlatanovic, Vesna Zivkovic, Milena Jankovic, Natasa Radosavljevic, Sinisa Ducic, Jovan Ducic, Jasna Stojkovic, Kristina Jovanovic, Dejan Nikolic

**Affiliations:** 1 Department for Physical Medicine and Rehabilitation, Faculty of Medicine, University of Niš, 18000 Niš, Serbia; draganzlatanovic1@gmail.com (D.Z.); petvesna67@gmail.com (V.Z.); 2 Clinic for Physical Medicine and Rehabilitation, University Clinical Center Niš, 18000 Niš, Serbia; 3 Faculty of Medicine, University of Belgrade, 11000 Belgrade, Serbia; milena.jankovic.82@gmail.com (M.J.); sinisaducic@gmail.com (S.D.); jovanducic98@gmail.com (J.D.); stojkovic.jasna@yahoo.com (J.S.); denikol27@gmail.com (D.N.); 4 Neurology Clinic, University Clinical Center of Serbia, 11000 Belgrade, Serbia; 5 Department of Biomedical Sciences, State University of Novi Pazar, 36300 Novi Pazar, Serbia; dr.natasa.radosavljevic@gmail.com; 6 Department of Pediatric Surgery, University Children’s Hospital, 11000 Belgrade, Serbia; 7 Department of Physical Medicine and Rehabilitation, University Children’s Hospital, 11000 Belgrade, Serbia; 8 Department of Pediatrics, University Children’s Hospital, 11000 Belgrade, Serbia; kristina.jovanovic104@gmail.com

**Keywords:** stroke, rehabilitation, motoric deficit, children

## Abstract

Pediatric stroke (PS) is an injury caused by the occlusion or rupture of a blood vessel in the central nervous system (CNS) of children, before or after birth. Hemiparesis is the most common motoric deficit associated with PS in children. Therefore, it is important to emphasize that PS is a significant challenge for rehabilitation, especially since the consequences may also appear during the child’s growth and development, reducing functional capacity. The plasticity of the child’s CNS is an important predecessor of recovery, but disruption of the neural network, specific to an immature brain, can have harmful and potentially devastating consequences. In this review, we summarize the complexity of the consequences associated with PS and the possibilities and role of modern rehabilitation. An analysis of the current literature reveals that Constraint-Induced Movement Therapy, forced-use therapy, repetitive transcranial magnetic stimulation, functional electrical stimulation and robot-assisted therapy have demonstrated at least partial improvements in motor domains related to hemiparesis or hemiplegia caused by PS, but they are supported with different levels of evidence. Due to the lack of randomized controlled studies, the optimal rehabilitation treatment is still debatable, and therefore, most recommendations are primarily based on expert consensuses, opinions and an insufficient level of evidence.

## 1. Introduction

Pediatric stroke (PS) is a rare pediatric condition with an incidence of 2–13/100,000. From 1990 to 2013, the prevalence of PS has globally increased by 35%, with 40,000 cases of recorded ischemic PS in 2013 [1] and with a long-term assessment of global tendency growth in the next 30 years [2].

According to the time of appearance, PS is defined as perinatal stroke (occurring from 20 weeks of gestation to the first 28 days after delivery) and childhood stroke (occurring from 29 days to 18 years of age) [3]. The incidence of perinatal stroke among live births is 1/1100 [4], and the incidence of childhood stroke is from 1.3 to 13 in 100,000 children [5].

**Figure 1:**
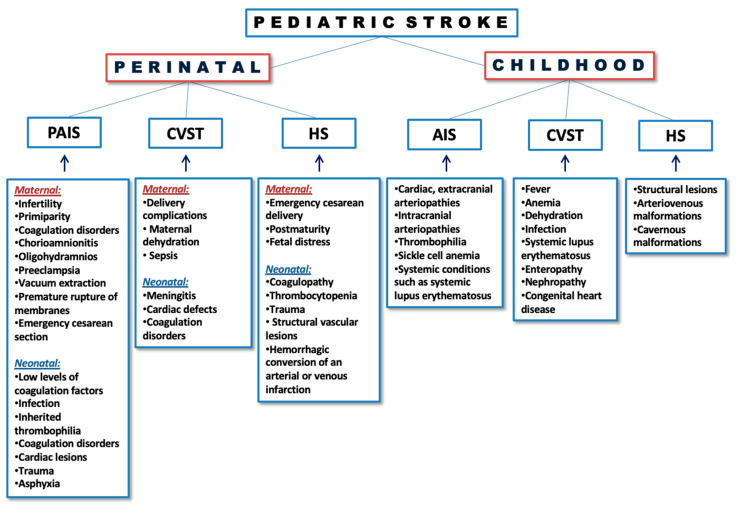
Pediatric stroke classification and specific risk factors. PAIS/AIS—(perinatal) arterial ischemic stroke; CVST—cerebral sinus venous thrombosis; HS—hemorrhagic stroke.

According to the mechanism by which stroke occurs, PS is classified into arterial ischemic stroke (AIS), cerebral sinus venous thrombosis (CVST) and hemorrhagic stroke (HS) [6] (Figure 1). The most represented mechanism of PS is AIS, which can be defined as a brain or spinal cord injury caused by a lack of oxygen in the affected area. Usually, AIS is the consequence of blood flow obstruction caused by blood clots. In patients with AIS, the area that is vascularized by the middle cerebral artery is most commonly affected [6,7]. CVST affects predominantly younger populations with variable etiology, clinical presentation and prognosis. In CVST, two pathological mechanisms are described: cerebral vein occlusion and venous sinus occlusion [8]. According to deVeber et al., the diagnostic criterion for AIS in infants and children older than 1 month is the sudden onset of a focal neurological deficit that can be transient or permanent in duration. In neonates with AIS and children of all ages with CVST, the criteria are seizures, lethargy or a focal neurological deficit [9]. HS is defined as a nontraumatic intracerebral hemorrhage (ICH), nontraumatic subarachnoid hemorrhage or intraventricular hemorrhage in a full-term neonate or child. Common presentation of ICH involves acute symptomatic seizures both in perinatal (60%) and childhood (36%) periods [6,10].

Pediatric stroke leads to long-term motoric deficiency and invalidity among children [11]. Rehabilitation is the core of treatment among children living with the consequences of PS. The role of rehabilitation remains irreplaceable, primarily due to the lack of early stroke diagnosis. Secondly, hyperacute recanalization treatment options, such as thrombolysis or mechanical thrombectomy, are rarely used and applied in the pediatric population [12]. In a retrospective case series study from several hospitals in Beijing that included 312 children with AIS and HS aged between 1 month and 18 years, none of 172 patients with AIS received hyperacute treatment (thrombolysis and mechanical thrombectomy). In this group of patients, 17.44% received antithrombocites for acute treatment, and 5.23% received anticoagulants [13].

## 2. Etiology of Pediatric Stroke

### 2.1. Perinatal Stroke—Risk Factors

Risk factors associated with perinatal arterial ischemic stroke are divided into maternal and neonatal risk factors. Maternal factors include infertility, primiparity, coagulation disorders, chorioamnionitis, oligohydramnios, preeclampsia, vacuum extraction, premature rupture of membranes and emergency cesarean section [14]. Neonatal factors include normal activation of coagulation factors in the mother and low levels of factors in the infant just before and after the time of delivery, infection, inherited thrombophilia, coagulation disorders, cardiac lesions, trauma and asphyxia [15].

Risk factors associated with CVST are not well established yet, but delivery complications, maternal dehydration, sepsis, meningitis, cardiac defects and coagulation disorders may be considered potential risk factors [3,16].

The causes of perinatal HS among term infants are coagulopathy, thrombocytopenia, trauma and, rarely, structural vascular lesions. Although they cannot be considered immediate causes, they are more commonly observed among male newborns after emergency cesarean delivery, following postmaturity and with fetal distress [3,17] (Figure 1).

### 2.2. Childhood Stroke—Risk Factors

The cause of AIS in childhood is most commonly multifactorial. The causes can be divided into cardiac, extracranial arteriopathies, intracranial arteriopathies, thrombophilia, sickle cell anemia and systemic conditions, such as systemic lupus erythematosus [18].

CVST in childhood often occurs as a result of several age-related risk factors in the formation of thrombus: fever, anemia, dehydration, infection, systemic lupus erythematosus, enteropathy, nephropathy, congenital heart disease, etc. [3].

In approximately 75% of cases, HS in childhood is caused by structural lesions [3]. A retrospective study conducted in China revealed that the main causes of acute ischemic stroke were cerebrovascular diseases, especially moyamoya (68.6%), and the leading cause of hemorrhagic stroke was arteriovenous malformations (51.43%) and cavernous malformation (20%) [13].

## 3. Specific Features of Pediatric Stroke

### 3.1. Motor Deficits following Pediatric Stroke in the Pediatric Population

**Figure 2:**
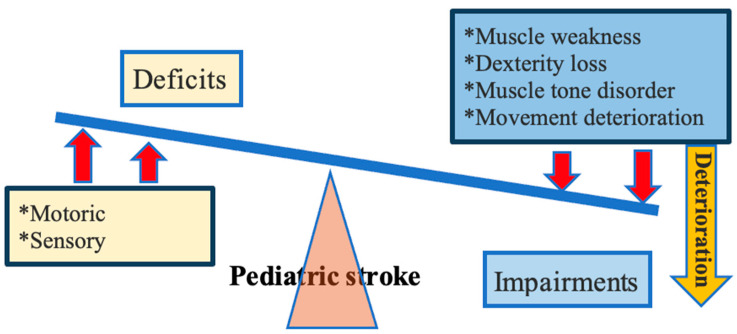
Deficits and impairments in pediatric stroke.

Pediatric stroke often leads to lifelong motoric, cognitive, communicational or sensory deficits [11]. In clinical presentation, motoric deficits and/or associated disorders are predominantly observed. Deficiencies may present as a weakness of one arm and/or one leg (in the case of hemiplegia) or weakness of both arms and/or both legs (in the case of quadriplegia or triplegia) [19]. Impairments vary and may include muscle weakness as well as a loss of dexterity, muscle tone disorders and deterioration of the quality and coordination of movements. As motoric and sensory deficits are more severe, impairments are more challenging in patients with pediatric stroke (Figure 2). Even though typical presentation involves hemiparesis, deficits are not exclusively unilateral, especially in the case of hemorrhagic stroke [20]. According to the literature data, the most common type of pediatric stroke is acute ischemic stroke, and the outcomes of this condition are the most studied ones [6].

#### 3.1.1. Perinatal Stroke

Approximately 80% of cases of perinatal stroke are of the arterial ischemic etiology [3]. Aside from etiology, there are other classifications of perinatal stroke. Based on the time of diagnosis, it is divided into acute and retrospective, and another classification is according to the age at presentation (fetal, preterm neonatal, term neonatal and stroke in infancy/childhood) [21].

The acute phase of perinatal stroke in the fetal period is asymptomatic, and there are no characteristic features. Premature infants may be asymptomatic, but apnea, bradycardia, desaturation, seizures and encephalopathy may occur as well [22]. Term infants, on the other hand, are symptomatic, with encephalopathy and the clinical presentation of seizures. PS commonly remains unrecognized in the acute phase since it does not manifest with signs of an acute motor deficit, as it is usually in the adult population, and due to its delayed presentation in early childhood or later, it is called presumed PS [3,21]. Presumed PS usually presents with hemiparesis after the first month following delivery, and it is classified based on the characteristic imaging findings as either arterial presumed perinatal ischemic stroke (APPIS), presumed perinatal hemorrhagic stroke (PPHS) or periventricular venous infarction (PVI), which is a presumed fetal stroke [21]. Newborns have no motor deficits, and motor asymmetry or early hand preference is observed at the child’s age of four to six months [23]. Perinatal stroke is the leading cause of hemiparetic cerebral palsy (CP), and the clinical presentation of CP for the aforementioned reason may therefore precede the diagnosis of PS. Therefore, it is essential to emphasize the importance of the early diagnosis of PS by assessing general motor activity in newborns that are symptomatically or anamnestically at risk [24].

With limited options for prevention and intervention, neurorehabilitation is the focus of improvement outcomes [21].

#### 3.1.2. Childhood Stroke

Compared to perinatal stroke, childhood stroke leads to higher rates of motor deficits [6]. The period between the 28th day and the 1st year of a child’s life is particularly vulnerable in terms of the consequences related to PS [18]. This is the time frame when PS causes the highest prevalence of moderate and severe forms of motor deficit, and this trend decreases with the child’s age. Among older age groups, no differences are observed in the degree of recovery of motor functions, which, according to the authors of the International Pediatric Stroke Study Group [25], implies that recovery mechanisms are common for all ages or that the brains of the youngest ones are characterized with concomitant contradictory processes, characterized by plasticity in one hand and vulnerability in the other one. The current literature has not provided any conclusion regarding this dilemma yet.

Hemiplegia is the most frequent acute clinical manifestation of AIS in childhood, with a frequency of 72–90% [26], and the prevalence of chronic hemiplegia in the literature varies from 12% [27] to 56% [28]. Studies focusing on long-term motor outcomes after childhood stroke have reported persisting hemiplegia in 44% to 66% of cases [29,30].

Compared to AIS, HS is reported to have better motor and functional outcomes. The results of a comparative study revealed that, in 60% of children with HS, rehabilitation led to a 4.5-month-long average duration of neurological deficit following the insult, compared to 89% of children with AIS. During the same follow-up period, 64% of children with HS regained functional use of their arm, compared to only 42% of children with AIS [31].

Compared to AIS, CVST seems to have lower long-term rates of disability. Normal neurological outcomes were reported in 39–63% of patients, whereas disability was observed in 18–42% of patients with pediatric CVST [6].

### 3.2. Specificity of the Child’s Brain Recovery after Pediatric Stroke—Advantage or Disadvantage

After a cerebrovascular insult, there are two possible outcomes regarding the brain recovery process: behavioral restitution and compensation. Behavioral restitution is a recovery process resulting in the return of normal patterns of motor control, i.e., recovery to the state before the insult occurred. This mechanism represents the complete recovery of damaged neural networks. Compensation is a recovery process in which patients adopt new motor patterns as a substitute for those they can no longer perform through neural learning circuits. These mechanisms are common for children as well as adults [32].

Evidence regarding recovery outcomes between children and the adult population is conflicting [33]. Research results have not yet provided an unambiguous answer. As we previously commented, disputing some views of better recovery of children’s brains, 66% of children with childhood stroke had long-term motor and functional deficits [30,31]. Greenham et al. supported such conclusions, stating that children’s brains do not recover better than adults [34]. Contrary to the conclusion of Srivastava et al. [21], outcomes specific to the developing brain, associated with increased neuroplasticity, result in a child’s ability to walk after a stroke, which is not a characteristic of adults with a similar lesion in the central nervous system. Therefore, in the context of this dilemma, it is important to emphasize that patterns and trajectories of recovery differ between children and adults. The increased capacity for the neuroplasticity of the brain relates to the specificity of the child’s brain, but an interruption of the neural network by a stroke can have harmful consequences that are specific to the immature brain [35]. Unlike the population of adults, the child’s brain goes through restructuring after PS. Prenatally, brain development mainly consists of neurogenesis and neuronal migration, and postnatally, in order to arrange mature neuronal networks, dominant patterns include glial cell proliferation, integration and synaptic development. As indicated by many genes and cellular processes, which are reactivated after the insult and are typical for early stages of neurodevelopment, recovery after stroke recapitulates developmental programs, and this is one point of view [35,36]. However, other studies’ results have so far shown significant differences in gene expression after an insult between an immature brain and the brain of an adult [37,38,39]. The fact that the brain quadruples in size during preschool age leads us to the assumption that, in children, natural growth and development may provide a longer recovery period [40]. Unfortunately, correlations between PS, the neuroplasticity of the developing brain and recovery remain insufficiently scrutinized [39].

### 3.3. Assessment of the Specificity of Motor and Functional Deficit after Pediatric Stroke

Aside from insufficiently explored mechanisms of recovery of the child’s brain following an insult, measures of therapeutic monitoring and outcome impose an additional confusion and challenge.

The core of this problem lies in different validated tests that recognize one or both previously mentioned recovery mechanisms in a manner in which the results of examination regarding the same subject do not always logically correlate.

The Pediatric Stroke Outcome Measure (PSOM) is the most widely used scale for the assessment of PS outcomes [41]. There are two validated versions: one for children aged ≤2 years of life and one for children aged 2–16 years. The PSOM recognizes compensatory as well as behavioral mechanisms of restitution as a corresponding model. Therefore, it can be concluded that, from the aspect of rehabilitation, the PSOM can overestimate the overall performance of rehabilitation [42].

The Fugl-Meyer assessment [43] and the Modified Rankin Scale (mRS) [44] are used for the long-term follow up of recovery following PS since they are age-limited. The Fugl-Meyer assessment appraises the recovery of muscle activity throughout individual joint movements that do not involve synergy. The Modified Rankin Scale (mRS) is used to assess disability, and it estimates a patient’s ability to perform activities of daily life throughout rehabilitation using adopted compensatory mechanisms.

The definition of disability refers to the loss of motor function, which ultimately leads to a limitation or lack of ability to perform daily activities or to participate in real-life situations [45]. The child’s functional recovery is the focus of motor function rehabilitation after PS. The PSOM unfortunately does not provide this type of evaluation. Hence, in 2007, the World Health Organization adopted the International Classification for Functioning, Disability and Health (ICF-CY) in order to assess the health and disability of children and youth [45]. This classification defines the biopsychosocial aspect of disability, i.e., it assesses mutual interferences between the child’s health condition, social environment and individual aspects. The WeeFIM [46], Assisting Hand Assessment [47] and the Canadian Occupational Performance Measure [48] are used to assess disability in children after PS.

During a child’s growth, PS thwarts the child’s functional independence, and this is a substantial difference between adults and children. Depending on the age, children with perinatal stroke, as well as younger children with childhood stroke, exhibit a motor deficit during growth that usually lasts for several years. Sophisticated interaction between neuro-developmental processes and neural injuries culminates with the clinical appearance of certain neurological deficits as well as improvements [35]. Thus, delayed acquirement of developmental milestones is characteristic for younger children, whereas older children might suffer from the loss of acquired motor functions, just like adults. Studies have concluded that preschool and school-aged children had improved gross motor functions during the first year after PS, but not fine motor skills [27]. During the same period of time, contrary to preschool and school-aged children, newborns had developed deficits [27]. The late onset of motor deficits during the child’s neurodevelopment requires a longer follow-up period, since the consequences of PS may otherwise be overlooked. In support of this claim, a multicenter Canadian registry reported moderate to severe deficits in 32% of patients after PS in a group of young adults [49].

Motor deficit always negatively interferes with the child’s motor development and the acquisition of new abilities during the child’s growth. Furthermore, it is a fact that children after PS, during the time of growth and the acquisition of motor functions, “acquire” disorders in several domains of the ICF [34,50]. By examining children’s participation in activities of daily life, after AIS in childhood, a study conducted by Simon-Martinez et al. enlightened a much wider problem related to the consequences associated with PS [50]. This was the first study to investigate children’s limitations after AIS and their relationship with ICF-CY components. This study included children previously diagnosed with AIS, with or without hemiparesis, and indicated that manual ability was reduced in both groups. Such conclusions imply that even children who do not have hemiparesis associated with PS will eventually have difficulties related to the use of their upper limbs in their daily-life activities, which will ultimately affect their overall quality of life. These devastating results and conclusions point in the direction of a so far unrevealed dimension of problems affecting children who are living with the consequences of PS, and they open many new dilemmas. The logical dilemma refers to the possibilities and domains of rehabilitation, i.e., the importance of long-term, regular monitoring of all children dealing with PS. The following questions arise:

(a) What is the optimal follow-up time for children after PS?
(b) Does behavioral restitution mean complete recovery for the child?
(c) What is the optimal set of tests and scales that evaluate all outcomes?

## 4. Rehabilitation of Motor and Functional Deficits of Children after Pediatric Stroke

Almost all aspects of stroke, including incidence, etiology, clinical presentation and recovery in adults, differ from the pediatric population, especially in terms of the specificities of the adults’ and children’s CNS during the damage and recovery phases [38,51]. Despite all these differences, therapeutic protocols for children are primarily based on the extrapolation of stroke in adults [52]. However, improvements and important advances in evidence-based PS research and therapy have been made in recent years. Nevertheless, pediatricians have an insufficient level of awareness regarding PS, which results in a late diagnosis, insufficient experience in the application of acute antithrombotic or anticoagulant treatment as well as secondary prevention, including thrombolysis or thrombectomy. Consequently, children with PS remain deprived of modern therapeutic treatment protocols and options [13].

### 4.1. Current Recommendations

**Figure 3:**
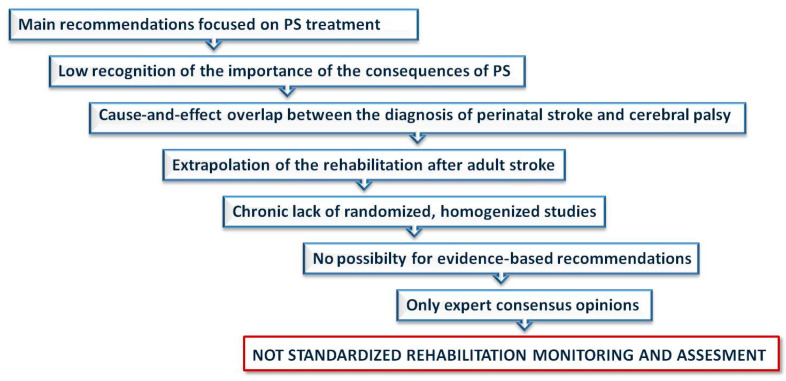
Current rehabilitation challenges after pediatric stroke (PS).

In the current literature, there are insufficient data regarding evidence-based pediatric rehabilitation following PS. Studies are mainly based on recommendations. The aforementioned differences indicate the necessity for evidence-based rehabilitation of children after PS (Figure 3). There are Canadian [53], Australian [54] and United Kingdom [20] guidelines for rehabilitation after PS. The American Heart Association (AHA) and the American Stroke Association (ASA) support Ferriero DM et al. and published the Management of Stroke in Neonates and Children, which is considered an American guideline [3].

An illustrative example of the previously stated claim that children’s rehabilitation is based purely on recommendations is the Australian clinical consensus guideline for the subacute rehabilitation of childhood stroke [54]. It refers to the strategy of subacute rehabilitation treatment that is specific to each domain associated with childhood stroke, and it provides a framework for rehabilitation therapy. In cases where evidence is inadequate or absent, a modified Delphi process might be applied to develop consensus-based recommendations. These guidelines contain 56 recommendations, of which only 1 is evidence-based, and 55 are consensus recommendations. In the introductory part, the authors point out that there is significant evidence that an individual, interdisciplinary approach to rehabilitation after a brain insult among adults improves the outcome, but there is no such evidence in children. The lack of research consequently led to a lack of evidence in Australia, which ultimately resulted in the lack of standardization of subacute rehabilitation of children with childhood stroke. When asked which interventions improve the outcome of motor deficits in children with stroke, experts managed to provide answers only according to consensus opinions. Only three papers met the criteria regarding different levels of evidence, and their results were taken into consideration with the aim of making recommendations based on evidence. The results of Kirton et al. [55] provide preliminary evidence that repetitive transcranial magnetic stimulation (rTMS) improves the grip strength of impaired upper limbs after childhood stroke. Modified Constraint-Induced Movement Therapy (mCIMT) of hands in children with hemiparesis after AIS raised satisfactory rates regarding therapy among parents and children, but unfortunately without any improved objective parameters [56]. The shortcoming of both studies is that they were conducted on small samples. A study [57] conducted on a larger sample investigated the effect of Proprioceptive Neuromuscular Facilitation (PNF) in improving muscle strength, but the inadequate analysis of the results imposes certain limitations for the generalization of the conclusions. Therefore, due to the poor quality of the evidence in all three studies, evidence-based recommendations could not be implemented, but the recommendations were based on the clinical experiences and expertise of the authors of the Australian guideline. The recommendations for the subacute rehabilitation of motor function disorders after childhood stroke are as follows: therapy is directed toward the goals determined by the child, parent and the therapist; the child’s active participation in motor learning is incorporated through repetition and intensive exercise (more than twice per week); and bimanual therapy should be considered.

The 2016 guidelines for rehabilitation after stroke were published as part of the Canadian stroke best practice recommendations, and they included PS recommendations as well [53]. In 2020, an updated version of the Canadian recommendations was released, but PS rehabilitation guidelines were unfortunately not included [58].

Regarding the management of stroke in neonates and children, a scientific statement from the American Heart Association/American Stroke Association [3] primarily refers to stroke treatment in neonates and children. Rehabilitation following AIS is recommended in a form of general recommendations: age-adjusted rehabilitation and the long-term follow up of children after PS, including a recommendation that constraint-induced movement therapy should be considered in cases of unilateral hand dysfunction after AIS in childhood. After ischemic neonatal stroke, Goals Activity Motor Enrichment has provided promising results so far.

Regarding the rehabilitation of motor deficits following PS, the most specific and detailed guidelines were released by the United Kingdom—Royal College of Paediatrics and Child Health in terms of rehabilitation modalities, but their drawback is that they are based on studies that include a mixed clinical population of children with PS, CP and acquired brain injuries [20]. They are limited to childhood stroke, and they recommend rehabilitation without specifying the exact rehabilitation protocol. Evidence supporting traditional neurodevelopmental therapy (NDT) for pediatric rehabilitation in neurological conditions is weak. Motor interventions that may be applicable to child stroke rehabilitation include Constraint-Induced Movement Therapy (CIMT), bi-manual therapy, Electromyography (EMG), triggered neuromuscular stimulation (NMS), functional electrical stimulation (FES), robot-assisted interactive therapy and virtual reality. The application of botulinum toxin in the spastic musculature of the upper extremities is recommended in combination with occupational therapy. Botulinum toxin application in the case of spastic musculature of the lower extremities due to central motor neuron lesions in children with CP has undeniably proven to be effective [59,60,61]. The lack of randomized, homogenized studies is the evident reason why the recommendations are applicable only for the upper extremities, since the mechanism of spasm formation is identical in children after PS and in children with a spastic form of CP. Therefore, our efforts must be addressed to provide evidence-based recommendations.

### 4.2. Evidence-Based Methodology and Rehabilitation of Motor and Functional Deficits of Children after Pediatric Stroke

According to the methodology of scientific and professional research, an interview is the first and most important part of research. The fact that motor deficits become apparent during the child’s growth after PS imposes some logical question to which we do not yet have answers:

- Should neurodevelopmental treatment be applied only to babies who have a motor deficit or also to those who have an anamnestic risk and have normal neurokinesiology findings?

In the literature, the lack of a homogenized group of patients with PS in the context of the success of evidence-based rehabilitation is evident based on a literature search for last 10 years. The search strings that were used included pediatric stroke, arterial ischemic stroke, cerebral sinus venous thrombosis, hemorrhagic stroke, child, childhood, motoric deficit, disability, rehabilitation, physical therapy and outcome. The defined search terms were analyzed in further databases: PubMed, Medline, Scopus and Google Scholar. The search results identified only two reviews [11,62].

Mirkowski et al. published a review paper in 2019, and they evaluated the success of the rehabilitation of motor and cognitive deficits after PS, supported by evidence [62]. The research included children with perinatal and childhood stroke in the period from 1980 to 2017. Out of 4602 studies, only 18 met the criteria that 50% of participants in the study had PS and that the study was conducted on a group of three or more participants, which is devastating data, indicating how long PS has been unrecognized as a significant cause of disability in children. The success of the rehabilitation of motor deficits was assessed in 15 papers, with 14 papers related to the rehabilitation of the upper extremities and only 1 related to the rehabilitation of the lower extremities. CIMT, forced-use therapy, rTMS, FES and robot-assisted therapy have shown improvements in all or some hand motor outcomes in children with hemiparesis or hemiplegia after stroke, supported by varying levels of evidence. The application of transcranial direct current stimulation (tDCS) did not result in motor and functional improvements in hemiparesis of the upper extremities in children after an insult.

Studies evaluating the improvement of the activity of the affected limb by applying CIMT during rehabilitation, which lasts 3 to 6 months after therapy, were the most common ones [56,63,64,65,66]. Rehabilitation success ranged according to the ability of using hands in daily-life activities, but there are less data regarding the overall quality of movements. Therefore, no precise answer was given regarding whether recovery was due to a compensatory mechanism or whether biological restitution was achieved.

The combination of CIMT and rTMS has shown a positive effect six months after rehabilitation. rTMS in combination with motor learning therapy, as well as the combination of CIMT and motor learning therapy, can improve the function of the upper limbs, and therefore, the mentioned combined therapies are recommended with a 1b level of evidence [63,67].

The application of non-invasive rTMS gives promising results at a 1b level of evidence regarding the prognosis of recovery and rehabilitation of the upper extremities of children after PS.

The success of the rehabilitation of the motor deficit of lower extremities through walking training indicated a potential therapeutic benefit [68].

After a long-term scientifically unfounded approach to the rehabilitation of children after PS, based on the principle of extrapolation of the rehabilitation of the adult population after a stroke, another misguiding approach is the assessment of the success of rehabilitation treatments of a heterogeneous pediatric population with CP, PS and brain injury. One of the important reasons is the cause-and-effect overlap between the diagnosis of perinatal stroke and CP. The diagnosis of CP includes, in addition to motor deficits, a much wider range of disorders and activity limitations, unlike PS. The logical dilemma is whether current modern rehabilitation protocols for children with CP are optimal for PS, and an additional dilemma refers to the hypothesis of whether rehabilitation in the pediatric population with PS is based on the extrapolation of children with CP. In a review paper, Hart et al. [11] assessed the success of the rehabilitation of motor deficits based on ICF categories. Of the 3735 studies, 16 were taken into consideration. During a four-year timeframe, 16 papers met the specified criteria (stroke was more accurately defined, neuromotor treatment was described, and the pediatric population was in focus). This review paper was the first to focus exclusively on PS and neuromotor treatment, with the aim of providing an answer from the available, contemporary, expert literature on evidence-based neuromotor treatment. However, the number of papers that meet the inclusion criteria is still small, which clearly indicates a chronic lack of homogenized, randomized studies. Neuromotor treatments were organized according to the ICF framework categories of body structure function (BSF), activity, participation and environment. Another fact, that there is no ICF Core Set for pediatric stroke, in contrast to CP, indicates the lack of recognition of the importance of the consequences of PS by the World Health Organization. The Body Structures and Functions category was the focus of all works, from different aspects. Robot-assisted upper extremity motor practice, exoskeletons and the application of botulinum toxin to spastic musculature have given results showing increases in the range of motion and muscle strength of the upper extremities. The combined application of rTMS with CIMT has given better results than the application of CIMT alone. The novelty is that combining tDCS with CIMT or occupational therapy has resulted in a positive outcome, whereas the application of tDCS alone has not proven efficient, and it was not recommended by Mirkowski et al. [62]. The application of FES and neuromuscular electrical stimulation (NMES), on the other hand, was supported. Research including neurorehabilitation with robot-assisted therapy and virtual game therapy has become more favored. The results favor the combined administration of botulinum toxin and modified Constraint-Induced Movement Therapy (mCIMT), compared to the administration of botulinum toxin and intensive conventional therapy. The function of the upper extremity was improved in all previously mentioned rehabilitation treatments. The quantitative indicators of the results of hand promotion therapy, which parents implement at home in children aged up to 6 months, has not given positive results. In the domain of activity, the recommended therapies are mCIMT and CIMT, independently or in combination with robotic bimanual training or hand arm bimanual intensive training following CIMT intervention. In the review of the literature, another problem was observed: a regression to the correlation of the target domain of applied therapies and outcome measures, with a recommendation to apply expansive outcome measures [11].

In the correlation to all previously mentioned modalities of motor deficit rehabilitation following PS, robot-assisted therapy is increasingly prevalent, as it represents the most innovative therapy and, due to its characteristics, it can be individually adapted to each child. This therapy easily motivates children to actively participate in performing movements that simulate daily-life activities, with a very important feedback loop that incorporates sight, hearing and proprioperception [69]. While evaluating the success of therapy related to the robot-assisted neurorehabilitation of the hand, we observed great diversity in the use of tests and scales: the Assisting Hand Assessment (AHA) [47], Pediatric Motor Activity Log (PMAL) [70], ABILHAND-Kids [71], Wolf Motor Function Test [72] and Test of Arm Selective Control (TASC) [73], among others. An arising dilemma is whether the authors resort to specific tests in the study design. From which test do they expect the best results, and what could promote a certain model of robot-assisted neurorehabilitation? Therefore, many questions and dilemmas remain open. The most important ones are related to the exact definition of the protocols, the optimal duration of therapy, the measure of the rehabilitation outcome and whether the use of robot assistance reduces the duration of rehabilitation and improves the final outcome. Current knowledge indicates that the application of robot-assisted therapy cannot replace the usual individual exercise techniques in children, but it has been proven to contribute to functional recovery [62].

## 5. Conclusions

For many years, the rehabilitation of children with PS was based on the extrapolation of the rehabilitation of adults after stroke. Recently, an impression has been gained that rehabilitation protocols implemented on heterogenous groups of children with CP and brain injuries can be administered on pediatric patients with PS.

Due to the lack of awareness of PS in professional practice, as a disease in expansion, the choice of rehabilitation modality depends on board-certified physical medicine and rehabilitation specialists. The impression is that the trend of the implemented rehabilitation of children with PS is based on the extrapolation of the rehabilitation protocols for children with CP. In homogenous studies of children with PS, there is evident representation of scientific papers on the rehabilitation of motor deficits of the upper extremity, and research studies of rehabilitation for lower extremities are so far insufficient. 

The increasingly prevalent PS, along with the opening of the “black box” of the rehabilitation of children after PS, should focus future research on clearly defining protocols, with adopted outcome measures for each therapeutic modality or motoric deficit.

Our work points to the lack of randomized and homogenized studies as the main reason why many physical therapy and rehabilitation modalities are not yet recommended or not supported by evidence.

## Data Availability

The data presented in this study are available on reasonable request from the corresponding author.

## References

[1] Krishnamurthi R.V., Deveber G., Feigin V.L., Barker-Collo S., Fullerton H., Mackay M.T., O′Callahan F., Lindsay M.P., Kolk A., Lo W. (2015). GBD 2013 Stroke Panel Experts Group. Stroke Prevalence, Mortality and Disability-Adjusted Life Years in Children and Youth Aged 0–19 Years: Data from the Global and Regional Burden of Stroke 2013. Neuroepidemiology.

[2] Wafa H.A., Wolfe C.D.A., Emmett E., Roth G.A., Johnson C.O., Wang Y. (2020). Burden of Stroke in Europe: Thirty-Year Projections of Incidence, Prevalence, Deaths, and Disability-Adjusted Life Years. Stroke.

[3] Ferriero D.M., Fullerton H.J., Bernard T.J., Billinghurst L., Daniels S.R., DeBaun M.R., deVeber G., Ichord R.N., Jordan L.C., Massicotte P. (2019). Management of Stroke in Neonates and Children: A Scientific Statement from the American Heart Association/American Stroke Association. Stroke.

[4] Dunbar M., Mineyko A., Hill M., Hodge J., Floer A., Kirton A. (2020). Population Based Birth Prevalence of Disease-Specific Perinatal Stroke. Pediatrics.

[5] Lynch J.K., Hirtz D.G., DeVeber G., Nelson K.B. (2002). Report of the National Institute of Neurological Disorders and Stroke workshop on perinatal and childhood stroke. Pediatrics.

[6] Malone L.A., Levy T.J., Peterson R.K., Felling R.J., Beslow L.A. (2022). Neurological and Functional Outcomes after Pediatric Stroke. Semin. Pediatr. Neurol..

[7] Roash E.S., Lo Warren D., Heyer Geoffrey L. (2012). Pediatric Stroke and Cerebrovascular Disorders.

[8] Alvis-Miranda H.R., Milena Castellar-Leones S., Alcala-Cerra G., Rafael Moscote-Salazar L. (2013). Cerebral sinus venous thrombosis. J. Neurosci. Rural Pract..

[9] deVeber G.A., MacGregor D., Curtis R., Mayank S. (2000). Neurologic outcome in survivors of childhood arterial ischemic stroke and sinovenous thrombosis. J. Child Neurol..

[10] Beslow L.A., Abend N.S., Gindville M.C., Bastian R.A., Licht D.J., Smith S.E., Hillis A.E., Ichord R.N., Jordan L.C. (2013). Pediatric intracerebral hemorrhage: Acute symptomatic seizures and epilepsy. JAMA Neurol..

[11] Hart E., Humanitzki E., Schroeder J., Woodbury M., Coker-Bolt P., Dodds C. (2022). Neuromotor Rehabilitation Interventions After Pediatric Stroke: A Focused Review. Semin. Pediatr. Neurol..

[12] Sotardi S.T., Alves C.A.P.F., Serai S.D., Beslow L.A., Schwartz E.S., Magee R., Vossough A. (2023). Magnetic resonance imaging protocols in pediatric stroke. Pediatr. Radiol..

[13] Deng Y., Liu G., Zhang G., Xu J., Yao C., Wang L., Zhao C., Wang Y. (2022). Childhood strokes in China describing clinical characteristics, risk factors and performance indicators: A case-series study. Stroke Vasc. Neurol..

[14] Lee J., Croen L.A., Backstrand K.H., Yoshida C.K., Henning L.H., Lindan C., Ferriero D.M., Fullerton H.J., Barkovich A.J., Wu Y.W. (2005). Maternal and infant characteristics associated with perinatal arterial stroke in the infant. JAMA.

[15] Nelson K.B., Lynch J.K. (2004). Stroke in newborn infants. Lancet Neurol..

[16] Fitzgerald K.C., Williams L.S., Garg B.P., Carvalho K.S., Golomb M.R. (2006). Cerebral sinovenous thrombosis in the neonate. Arch. Neurol..

[17] Cole L., Dewey D., Letourneau N., Kaplan B.J., Chaput K., Gallagher C., Hodge J., Floer A., Kirton A. (2017). Clinical Characteristics, Risk Factors, and Outcomes Associated with Neonatal Hemorrhagic Stroke: A Population-Based Case-Control Study. JAMA Pediatr..

[18] Felling R.J., Sun L.R., Maxwell E.C., Goldenberg N., Bernard T. (2017). Pediatric arterial ischemic stroke: Epidemiology, risk factors, and management. Blood Cells Mol. Dis..

[19] Svensson K., Walås A., Bolk J., Bang P., Sundelin H.E.K. (2022). Adverse motor outcome after paediatric ischaemic stroke: A nationwide cohort study. Paediatr. Perinat. Epidemiol..

[20] Royal College of Paediatrics and Child Health (2017). Stroke in Childhood: Clinical Guideline for Diagnosis, Management and Rehabilitation.

[21] Srivastava R., Mailo J., Dunbar M. (2022). Perinatal Stroke in Fetuses, Preterm and Term Infants. Semin. Pediatr. Neurol..

[22] Sorg A.L., von Kries R., Klemme M., Gerstl L., Weinberger R., Beyerlein A., Lack N., Felderhoff-Müser U., Dzietko M. (2020). Risk factors for perinatal arterial ischaemic stroke: A large case-control study. Dev. Med. Child Neurol..

[23] Lee J., Croen L.A., Lindan C., Nash K.B., Yoshida C.K., Ferriero D.M., Barkovich A.J., Wu Y.W. (2005). Predictors of outcome in perinatal arterial stroke: A population-based study. Ann. Neurol..

[24] Zlatanovic D., Čolović H., Živković V., Stanković A., Kostić M., Vučić J., Tošić T. (2022). The importance of assessing general motor activity in premature infants for predicting neurological outcomes. Folia Neuropathol..

[25] Felling R.J., Rafay M.F., Bernard T.J., Carpenter J.L., Dlamini N., Hassanein S.M., Jordan L.C., Noetzel M.J., Rivkin M.J., Shapiro K.A. (2020). International Pediatric Stroke Study Group. Predicting Recovery and Outcome after Pediatric Stroke: Results from the International Pediatric Stroke Study. Ann. Neurol..

[26] deVeber G., Roach E.S., Riela A.R., Wiznitzer M. (2000). Stroke in children: Recognition, treatment, and future directions. Semin. Pediatr. Neurol..

[27] Cooper A.N., Anderson V., Hearps S., Greenham M., Ditchfield M., Coleman L., Hunt R.W., Mackay M.T., Monagle P., Gordon A.L. (2017). Trajectories of Motor Recovery in the First Year After Pediatric Arterial Ischemic Stroke. Pediatrics.

[28] Ganesan V., Hogan A., Shack N., Gordon A., Isaacs E., Kirkham F.J. (2000). Outcome after ischaemic stroke in childhood. Dev. Med. Child Neurol..

[29] Christerson S., Strömberg B. (2010). Stroke in Swedish children II: Long-term outcome. Acta Paediatr..

[30] Hurvitz E.A., Beale L., Ried S., Nelson V.S. (1999). Functional outcome of paediatric stroke survivors. Pediatr. Rehabil..

[31] Yvon E., Lamotte D., Tiberghien A., Godard I., Mardaye A., Laurent-Vannier A., Agostini M.D., Chevignard M. (2018). Long-term motor, functional, and academic outcome following childhood ischemic and hemorrhagic stroke: A large rehabilitation center-based retrospective study. Dev. Neurorehabil..

[32] Levin M.F., Kleim J.A., Wolf S.L. (2009). What do motor “recovery” and “compensation” mean in patients following stroke?. Neurorehabil. Neural. Repair..

[33] Goeggel Simonetti B., Cavelti A., Arnold M., Bigi S., Regényi M., Mattle H.P., Gralla J., Fluss J., Weber P., Hackenberg A. (2015). Long-term outcome after arterial ischemic stroke in children and young adults. Neurology.

[34] Greenham M., Gordon A., Anderson V., Mackay M.T. (2016). Outcome in Childhood Stroke. Stroke.

[35] Malone L.A., Felling R.J. (2020). Pediatric Stroke: Unique Implications of the Immature Brain on Injury and Recovery. Pediatr. Neurol..

[36] Cramer S.C., Chopp M. (2000). Recovery recapitulates ontogeny. Trends Neurosci..

[37] López-Espejo M., Hernández-Chávez M. (2017). Prevalence and Predictors of Long-Term Functional Impairment, Epilepsy, Mortality, and Stroke Recurrence after Childhood Stroke: A Prospective Study of a Chilean Cohort. J. Stroke Cerebrovasc. Dis..

[38] Krägeloh-Mann I., Lidzba K., Pavlova M.A., Wilke M., Staudt M. (2017). Plasticity during Early Brain Development Is Determined by Ontogenetic Potential. Neuropediatrics.

[39] Felling R.J., Song H. (2015). Epigenetic mechanisms of neuroplasticity and the implications for stroke recovery. Exp. Neurol..

[40] Stiles J., Jernigan T.L. (2010). The basics of brain development. Neuropsychol. Rev..

[41] Kitchen L., Westmacott R., Friefeld S., MacGregor D., Curtis R., Allen A., Yau I., Askalan R., Moharir M., Domi T. (2012). The pediatric stroke outcome measure: A validation and reliability study. Stroke.

[42] Bulder M.M., Hellmann P.M., van Nieuwenhuizen O., Kappelle L.J., Klijn C.J., Braun K.P. (2011). Measuring outcome after arterial ischemic stroke in childhood with two different instruments. Cerebrovasc. Dis..

[43] Fugl-Meyer A.R., Jääskö L., Leyman I., Olsson S., Steglind S. (1975). The post-stroke hemiplegic patient. 1. a method for evaluation of physical performance. Scand. J. Rehabil. Med..

[44] Van Swieten J.C., Koudstaal P.J., Visser M.C., Schouten H.J., van Gijn J. (1988). Interobserver agreement for the assessment of handicap in stroke patients. Stroke.

[45] World Health Organization (2012). International Classification of Functioning, Disability and Health (ICF).

[46] Ottenbacher K.J., Msall M.E., Lyon N., Duffy L.C., Ziviani J., Granger C.V., Braun S., Feidler R.C. (2000). The WeeFIM instrument: Its utility in detecting change in children with developmental disabilities. Arch. Phys. Med. Rehabil..

[47] Krumlinde-Sundholm L., Holmefur M., Kottorp A., Eliasson A.C. (2007). The Assisting Hand Assessment: Current evidence of validity, reliability, and responsiveness to change. Dev. Med. Child Neurol..

[48] Cusick A., McIntyre S., Novak I., Lannin N., Lowe K. (2006). A comparison of goal attainment scaling and the Canadian Occupational Performance Measure for paediatric rehabilitation research. Pediatr. Rehabil..

[49] deVeber G.A., Kirton A., Booth F.A., Yager J.Y., Wirrell E.C., Wood E., Shevell M., Surmava A.M., McCusker P., Massicotte M.P. (2017). Epidemiology and Outcomes of Arterial Ischemic Stroke in Children: The Canadian Pediatric Ischemic Stroke Registry. Pediatr. Neurol..

[50] Simon-Martinez C., Kamal S., Frickmann F., Steiner L., Slavova N., Everts R., Steinlin M., Grunt S. (2021). Participation after childhood stroke: Is there a relationship with lesion size, motor function and manual ability?. Eur. J. Paediatr. Neurol..

[51] Ndondo A.P., Hammond C.K. (2022). Management of Pediatric Stroke—Challenges and Perspectives from Resource-limited Settings. Semin. Pediatr. Neurol..

[52] Tsze D.S., Valente J.H. (2011). Pediatric stroke: A review. Emerg. Med. Int..

[53] Hebert D., Lindsay M.P., McIntyre A., Kirton A., Rumney P.G., Bagg S., Bayley M., Dowlatshahi D., Dukelow S., Garnhum M. (2016). Canadian stroke best practice recommendations: Stroke rehabilitation practice guidelines, update 2015. Int. J. Stroke.

[54] Greenham M., Knight S., Rodda J., Scheinberg A., Anderson V., Fahey M.C., Mackay M.T., Victorian Subacute Childhood Stroke Advisory Committee (2021). Victorian Subacute Childhood Stroke Advisory Committee. Australian clinical consensus guideline for the subacute rehabilitation of childhood stroke. Int. J. Stroke.

[55] Kirton A., Chen R., Friefeld S., Gunraj C., Pontigon A.M., Deveber G. (2008). Contralesional repetitive transcranial magnetic stimulation for chronic hemiparesis in subcortical paediatric stroke: A randomised trial. Lancet Neurol..

[56] Gordon A., Connelly A., Neville B., Vargha-Khadem F., Jessop N., Murphy T., Ganesan V. (2007). Modified constraint-induced movement therapy after childhood stroke. Dev. Med. Child Neurol..

[57] Khalid S., Bashir M.S., Shah S.I., Noor R. (2015). Prognosis of stroke in children after three months of regular physical therapy in Lahore. J. Pak. Med. Assoc..

[58] Teasell R., Salbach N.M., Foley N., Mountain A., Cameron J.I., Jong A.D., Acerra N.E., Bastasi D., Carter S.L., Fung J. (2020). Canadian Stroke Best Practice Recommendations: Rehabilitation, Recovery, and Community Participation following Stroke. Part One: Rehabilitation and Recovery Following Stroke; 6th Edition Update 2019. Int. J. Stroke.

[59] Colovic H., Dimitrijevic L., Stankovic I., Nikolic D., Radovic-Janosevic D., Zivanovic D. (2014). The effects of botulinum toxin type A on improvement and dynamic spastic equinus correction in children with cerebral palsy—Preliminary results. Arch. Med. Sci..

[60] Colovic H., Dimitrijevic L., Stankovic I., Nikolic D., Radovic-Janosevic D. (2012). Estimation of botulinum toxin type A efficacy on spasticity and functional outcome in children with spastic cerebral palsy. Biomed. Pap. Med. Fac. Univ. Palacky Olomouc. Czech Repub..

[61] Colovic H., Dimitrijevic L., Stankovic I., Radovic-Janosevic D., Zlatanovic D. (2020). Botulinum toxin type A for spastic cerebral palsy: Is it time to change praxis?. J. Rehabil. Med..

[62] Mirkowski M., McIntyre A., Faltynek P., Sequeira N., Cassidy C., Teasell R. (2019). Nonpharmacological rehabilitation interventions for motor and cognitive outcomes following pediatric stroke: A systematic review. Eur. J. Pediatr..

[63] Kirton A., Andersen J., Herrero M., Nettel-Aguirre A., Carsolio L., Damji O., Keess J., Mineyko A., Hodge J., Hill M.D. (2016). Brain stimulation and constraint for perinatal stroke hemiparesis: The PLASTIC CHAMPS Trial. Neurology.

[64] Rickards T., Sterling C., Taub E., Perkins-Hu C., Gauthier L., Graham M., Griffin A., Davis D., Mark V.W., Uswatte G. (2014). Diffusion tensor imaging study of the response to constraint-induced movement therapy of children with hemiparetic cerebral palsy and adults with chronic stroke. Arch. Phys. Med. Rehabil..

[65] Taub E., Griffin A., Uswatte G., Gammons K., Nick J., Law C.R. (2011). Treatment of congenital hemiparesis with pediatric constraint-induced movement therapy. J. Child Neurol..

[66] Juenger H., Linder-Lucht M., Walther M., Berweck S., Mall V., Staudt M. (2007). Cortical neuromodulation by constraint-induced movement therapy in congenital hemiparesis: An FMRI study. Neuropediatrics.

[67] Eng D., Zewdie E., Ciechanski P., Damji O., Kirton A. (2018). Interhemispheric motor interactions in hemiparetic children with perinatal stroke: Clinical correlates and effects of neuromodulation therapy. Clin. Neurophysiol..

[68] Yang J.F., Livingstone D., Brunton K., Kim D., Lopetinsky B., Roy F., Zewdie E., Patrick S.K., Andersen J., Kirton A. (2013). Training to enhance walking in children with cerebral palsy: Are we missing the window of opportunity?. Semin. Pediatr. Neurol..

[69] Čolović H., Dimitrijević L., Đurić V., Janković S. (2020). Upper limb robotic neurorehabilitation after pediatric stroke. Srp. Arh. Celok. Lek..

[70] Taub E., Ramey S.L., DeLuca S., Echols K. (2004). Efficacy of constraint-induced movement therapy for children with cerebral palsy with asymmetric motor impairment. Pediatrics.

[71] Arnould C., Penta M., Renders A., Thonnard J.L. (2004). ABILHAND-Kids: A measure of manual ability in children with cerebral palsy. Neurology.

[72] Wolf S.L., Thompson P.A., Morris D.M., Rose D.K., Winstein C.J., Taub E., Giuliani C., Pearson S.L. (2005). The EXCITE trial: Attributes of the Wolf Motor Function Test in patients with subacute stroke. Neurorehabil. Neural. Repair..

[73] Sukal-Moulton T., Gaebler-Spira D., Krosschell K.J. (2018). The validity and reliability of the Test of Arm Selective Control for children with cerebral palsy: A prospective cross-sectional study. Dev. Med. Child Neurol..

